# An unusual presentation of biloma five years following cholecystectomy: a case report

**DOI:** 10.4076/1757-1626-2-8048

**Published:** 2009-09-10

**Authors:** Umashankkar Kannan, Rajinder Parshad, Subodh Kumar Regmi

**Affiliations:** 1 Department of Surgical Disciplines, All India Institute of Medical Sciences, Ansari nagar, New Delhi 110029, India

## Abstract

A 34-year-old female presented with right hypochondrial pain of 6 months following an uneventful open cholecystectomy about 5 years ago. A firm intra abdominal lump was felt in the right hypochondrium. Ultrasonography and computed tomography of the abdomen showed a large cystic lesion in relation to the porta hepatis. On exploration, a large cystic mass was found in relation to the undersurface of liver, adherent to the colon and duodenum. The cyst was excised leaving a cuff of cyst wall, densely adherent to the duodenum. A small opening with bile trickling through it was noted in the region of the confluence of hepatic ducts. Choledochotomy was done and T-tube placed. The bilious output from the sub-hepatic drain gradually decreased and the repeat T-tube cholangiogram on 14^th^ day following surgery was normal. The patient, at one year of follow-up is asymptomatic with normal liver function tests.

## Case presentation

**Figure 1:**
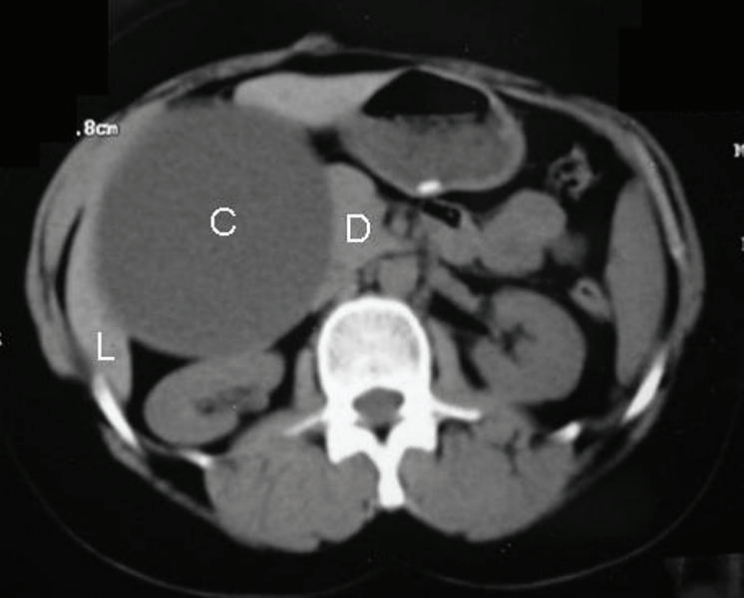
**Computed tomography of the abdomen showing a large cystic lesion (C) abutting the Liver (L) and the Duodenum (D)**.

A 34-year-old female of Asian origin presented with dull aching pain over the right hypochondrium, radiating to the back for 6 months with no history of fever, jaundice, hematemesis or malena. She had undergone an open cholecystectomy about 5 years back with an uneventful post operative period and was discharged on the tenth day following surgery. Rest of the history was unremarkable. On examination, a tender, firm intra abdominal lump of size 7 × 5 cm was felt in the right hypochondrium. The lump was extending to the lumbar region and was moving well with respiration. It was dull on percussion and continuous with liver dullness. Laboratory parameters were within normal limits except for an elevated serum alkaline phosphatase of 420 U/L (Normal 80- 240). Ultrasonography (US) of the abdomen showed a cystic lesion involving the segment V of the liver with a huge extra hepatic component along with small septations and internal debris. Contrast enhanced computed tomography (CECT) of the abdomen (Figure 1) showed a large well defined enhancing smooth walled cystic lesion in the right lobe of the liver adjacent to the porta hepatis. As the lesion was in close proximity to the first part of the duodenum a possibility of a duodenal duplication cyst was considered and an upper gastrointestinal endoscopy (UGIE) was performed. UGIE showed a deformed stomach with an extrinsic compression at the junction of first and second part of duodenum. Hydatid serology and 99 mTc Pertecnate Meckel's Scan for ectopic gastric mucosa were negative.

**Figure 2:**
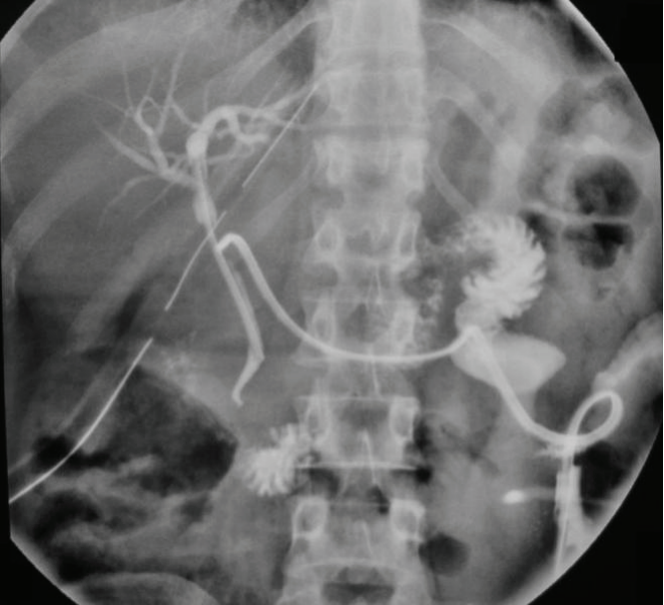
**T-Tube cholangiogram done on post operative day 14 shows normal biliary tract with patent bilio-enteric pathway**. Cholecystectomy was done about 5 years ago.

During the period of investigation, pain and the lump increased in size. The patient was explored through a right subcostal incision under general anesthesia. A large cystic mass of size 10 cm × 10 cm was found in relation to the undersurface of liver. The mass was adherent to the colon and duodenum and was overlying the region of porta hepatis. Aspiration of the cyst revealed bilious fluid. The cyst was excised leaving a cuff of cyst wall, which was densely adherent to the duodenum. Frozen section of the cyst wall showed only fibrocollagenous tissue. After excision of the cyst porta hepatis as exposed and an induration was noted in the region of the confluence of hepatic ducts with a small opening through which bile was trickling. The opening was cannulated and the cholangiogram was done to delineate the anatomy. Since biliary anatomy was not clear on intra operative cholangiography, opening was closed Choledochotomy done and a T tube placed in the bile duct. Abdomen was closed after placing a sub hepatic drain. In the post operative period, the drain output was bilious in nature. The drain output gradually decreased and finally stopped. A T-tube cholangiogram done on day 14 after surgery showed a normal bilio-enteric pathway with no leakage (Figure 2). The drain, the T-tube was then removed and the patient was discharged. At 1 year of follow-up is asymptomatic with normal liver function tests.

## Discussion

A biloma is a well-demarcated, encapsulated or not, bile collection outside the biliary tree secondary to iatrogenic, traumatic or spontaneous injury of the biliary tree. Gould and Patel used the term biloma for the first time in 1979 to describe a loculated collection outside the biliary tree but were then extended to include both intra and extra hepatic collections of bile. [1]. Leaking bile, by virtue of the detergent and tissue destroying action of the bile acids, elicits a low grade inflammation resulting in a thin capsule or in adhesions thereby forming an isolated collection called biloma.

Before the era of laparoscopy the rate of major bile duct injury for open cholecystectomy was about 0.1% and the total biliary injury rate was 0.1%. With laparoscopic technique the rates for major bile duct injury and the total biliary injury rate range from 0.3%-0.6% and 0.6%-1.5% respectively [2]. Bilomas resulting as a complication of cholecystectomy is often the leakage from an inadequately secured cystic duct stump, an accessory bile duct or a duct of Luschka in the gallbladder fossa of the liver.

Bilomas usually present with abdominal pain, nausea, anorexia, jaundice, fever and abdominal tenderness. The median time of diagnosis is usually 1-2 weeks [3]. The symptoms and signs may be subtle to the extent that in a series, the mean time of diagnosis was delayed up to 16.8 days [4]. Quite unusual of its kind, our case presented about 5 years following an uneventful open cholecystectomy and therefore it was not suspected as the cause of right hypochondrial lump. Literature review shows only one report of such a delayed presentation [5]. Probable reason for such a delayed presentation in our case could be a minor leakage following bile duct injury during cholecystectomy, which initiated a low grade inflammation and gradually walled off. The other hypothesized reasons reported for delayed presentations are low secretory pressure (< 30 cm of water) and low osmolality of the leaked bile [5].

The clinical history with the US, CT findings often establishes the diagnosis. Magnetic resonance cholangiopancreatography (MRCP) helps to find the site of leakage but useful only in the presence of an active leak. Percutaneous aspiration under US or CT guidance with biochemical test for Bilirubin confirms the diagnosis [6].

The appropriate treatment for biloma is drainage as everyone with undrained bile is at risk. Spontaneous reabsorption of bile collections larger than 4 cm is found to be rare and unpredictable [4]. Non-surgical methods constitute the treatment for majority of the cases. In the absence of a communication with the biliary tree, stricture or stone in the proximal biliary tree, percutaneous drainage with intravenous antibiotics achieves resolution of biloma [7]. Endoscopy identifies the extent and level of biliary injury and establishes if it can be treated endoscopically. Factors that predict favorable outcome of endotherapy for biliary leakage are: extra hepatic as opposed to intrahepatic lesions, injuries < 5 mm, distal obstruction that can be treated with sphincterotomy alone, and absence of bile peritonitis or intra abdominal abscess [8]. Endoscopic sphincterotomy (ES) and selective stent insertion resolves the leak in most of the cases. Surgical drainage is resorted only in the failure of percutaneous and endoscopic management.

## Conclusion

Bilomas must be considered in the differential diagnoses of abdominal cystic lesions even many years following surgery on the gallbladder and hepatobiliary system.

## Abbreviations

CECT: contrast enhanced computed tomography; ES: endoscopic sphincterotomy; MRCP: magnetic resonance cholangiopancreatography; UGIE: upper gastrointestinal endoscopy; US: ultrasonography.

## Consent

Written informed consent was obtained from the patient for publication of this case report and accompanying images. A copy of the written consent is available for review by the Editor-in-Chief of this journal.

## Competing interests

The authors declare that they have no competing interests.

## Authors' contributions

UK was a major contributor in writing the manuscript, RP made substantial contribution in conception, design and analysis of patient data. SKR contributed in conception and analysis of data.
